# A Unique Mechanochemical Redox Reaction Yielding Nanostructured Double Perovskite Sr_2_FeMoO_6_ With an Extraordinarily High Degree of Anti-Site Disorder

**DOI:** 10.3389/fchem.2022.846910

**Published:** 2022-03-16

**Authors:** Erika Tóthová, André Düvel, Ralf Witte, Richard A. Brand, Abhishek Sarkar, Robert Kruk, Mamoru Senna, Klebson Lucenildo Da Silva, Dirk Menzel, Vladimír Girman, Michal Hegedüs, Matej Baláž, Petre Makreski, Shiro Kubuki, Mária Kaňuchová, Jan Valíček, Horst Hahn, Vladimír Šepelák

**Affiliations:** ^1^ Institute of Nanotechnology, Karlsruhe Institute of Technology, Eggenstein-Leopoldshafen, Germany; ^2^ Institute of Geotechnics, Slovak Academy of Sciences, Košice, Slovakia; ^3^ Faculty of Physics and Center for Nanointegration Duisburg-Essen, University of Duisburg-Essen, Duisburg, Germany; ^4^ Faculty of Science and Technology, Keio University, Yokohama, Japan; ^5^ Department of Physics, State University of Maringá, Maringá, Brazil; ^6^ Institute of Condensed Matter Physics, Braunschweig University of Technology, Braunschweig, Germany; ^7^ Institute of Physics, Faculty of Science, P. J. Šafárik University, Košice, Slovakia; ^8^ Synthon s.r.o., Blansko, Czechia; ^9^ Institute of Chemistry, Faculty of Natural Sciences and Mathematics, Ss. Cyril and Methodius University in Skopje, Skopje, North Macedonia; ^10^ Graduate School of Science, Tokyo Metropolitan University, Tokyo, Japan; ^11^ Faculty of Mining, Ecology, Process Control and Geotechnologies, Technical University of Košice, Košice, Slovakia; ^12^ Faculty of Technology, College of Technology and Business in České Budějovice, České Budějovice, Czechia; ^13^ Faculty of Engineering, Slovak University of Agriculture, Nitra, Slovakia

**Keywords:** mechanochemistry, mechanochemical synthesis, double perovskite, anti-site disorder, Mössbauer spectroscopy

## Abstract

Strontium ferromolybdate, Sr_2_FeMoO_6_, is an important member of the family of double perovskites with the possible technological applications in the field of spintronics and solid oxide fuel cells. Its preparation *via* a multi-step ceramic route or various wet chemistry-based routes is notoriously difficult. The present work demonstrates that Sr_2_FeMoO_6_ can be mechanosynthesized at ambient temperature in air directly from its precursors (SrO, α-Fe, MoO_3_) in the form of nanostructured powders, without the need for solvents and/or calcination under controlled oxygen fugacity. The mechanically induced evolution of the Sr_2_FeMoO_6_ phase and the far-from-equilibrium structural state of the reaction product are systematically monitored with XRD and a variety of spectroscopic techniques including Raman spectroscopy, ^57^Fe Mössbauer spectroscopy, and X-ray photoelectron spectroscopy. The unique extensive oxidation of iron species (Fe^0^ → Fe^3+^) with simultaneous reduction of Mo cations (Mo^6+^ → Mo^5+^), occuring during the mechanosynthesis of Sr_2_FeMoO_6_, is attributed to the mechanically triggered formation of tiny metallic iron nanoparticles in superparamagnetic state with a large reaction surface and a high oxidation affinity, whose steady presence in the reaction mixture of the milled educts initiates/promotes the swift redox reaction. High-resolution transmission electron microscopy observations reveal that the mechanosynthesized Sr_2_FeMoO_6_, even after its moderate thermal treatment at 923 K for 30 min in air, exhibits the nanostructured nature with the average particle size of 21(4) nm. At the short-range scale, the nanostructure of the as-prepared Sr_2_FeMoO_6_ is characterized by both, the strongly distorted geometry of the constituent FeO_6_ octahedra and the extraordinarily high degree of anti-site disorder. The degree of anti-site disorder *ASD* = 0.5, derived independently from the present experimental XRD, Mössbauer, and SQUID magnetization data, corresponds to the completely random distribution of Fe^3+^ and Mo^5+^ cations over the sites of octahedral coordination provided by the double perovskite structure. Moreover, the fully anti-site disordered Sr_2_FeMoO_6_ nanoparticles exhibit superparamagnetism with the blocking temperature *T*_B_ = 240 K and the deteriorated effective magnetic moment *μ* = 0.055 *μ*_B_ per formula unit.

## Introduction

The straightforward synthesis of nanoscale and (simultaneously) far-from-equilibrium solids is a great challenge despite promising developments of synthesis techniques (Bensch and Breu, 2017). Nanostructured compounds with a nonequilibrium short-range structure can hardly be accessed by conventional (thermal) solid-state preparation routes requiring high synthesis temperatures. Also wet (solution) chemistry-based preparation ways lead to the thermodynamically stable rather than metastable products. Fortunately, such hurdles can be overcome by chemical reactions initiated or accelerated by the direct absorption of mechanical energy (Baláž et al., 2013; Šepelák et al., 2013; Wilkening et al., 2017; Boldyrev, 2018). In addition, the mechanically induced chemical reactions often outperform their conventional and solution-based counterparts in terms of sustainability (Bolm and Hernández, 2018; Michalchuk et al., 2021). As a consequence, the mechanically induced chemistry (mechanochemistry) becomes a “game-changer” (Gomollón-Bel, 2019), especially in chemical synthesis (Avvakumov et al., 2001; Friščić et al., 2020; Lapshin et al., 2021). Mechanochemistry is currently undergoing rapid expansion in a number of areas, and has been applied for the synthesis of various compounds, including energy storage materials (Gombotz and Wilkening, 2021; Schlem et al., 2021), pharmaceutics (Solares-Briones et al., 2021), metal-organic frameworks (Zhou et al., 2019; Głowniak et al., 2021), organic compounds (Andersen and Mack, 2018; Michalchuk et al., 2019), catalytic materials (Pickhardt et al., 2020; Porcheddu et al., 2020; Amrute et al., 2021), nanocomposites (Sherif El-Eskandarany et al., 2021), and nanocrystalline inorganic materials (Šepelák et al., 2012a; Lunghammer et al., 2019; De Oliveira et al., 2020). From the point of view of the type of mechanical impact, the strategies to mechanically promote a chemical reaction in matter include the use of mechanical milling, extrusion techniques, pulsed ultrasonication, and single-molecule force spectroscopy approaches, among others (Lapshin et al., 2021; O’Neill and Boulatov, 2021).

The mechanochemical synthesis (sometimes shortly called mechanosynthesis), studied in the present work is a reduction-oxidation (redox) reaction *via* high-energy ball milling of a stoichiometric mixture of SrO, α-Fe and MoO_3_ precursors at ambient temperature in air, leading to the formation of strontium ferromolybdate Sr_2_FeMoO_6_. Using the recently proposed pictographic nomenclature for mechanochemical syntheses (Michalchuk et al., 2021), the present redox reaction emphasizing the changes in valence state of the Fe and Mo cations can roughly be represented as 2 SrO + α-Fe^0^ + Mo^6+^O_3_ + ½ O_2_ →
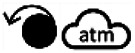
→ Sr_2_Fe^3+^Mo^5+^O_6_, where the symbols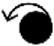 and 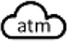
specify the reaction conditions; namely, they denote the use of “planetary ball mill” and “ambient atmosphere,” respectively. Although in the last few years a surge of investigations in the field of mechanochemistry has resulted in the mechanosynthesis of a variety of complex oxides by forcing a system to acquire metastable and non-equilibrium configurations (Zhang and Saito, 2012; Šepelák et al., 2013; Wilkening et al., 2017; Porodko et al., 2021; Szczȩśniak et al., 2021; Tsuzuki, 2021; Šepelák et al., 2021), there is no report in the literature on the nonconventional mechanochemical route to synthesize Sr_2_FeMoO_6_. Commonly, this material is prepared *via* a conventional multi-step ceramic route (Valenzuela et al., 2014; Kalanda et al., 2019; Alvarado-Flores et al., 2021) or various wet chemistry-based routes (Li et al., 2019; Farzin et al., 2020; Valdés et al., 2021); some of them involve a prolonged heat treatment at considerably high temperatures under reducing atmosphere (Iranmanesh et al., 2016). The present work demonstrates that Sr_2_FeMoO_6_ can be mechanosynthesized at ambient temperature in air directly from its precursors in the form of nanostructured powders, without the need for solvents and/or calcination under controlled oxygen fugacity, thus making the process very facile and efficient.

From the point of view of mechanochemistry, an important question providing a part of motivation for the present work is, whether it is possible to transform, by means of a mechanochemical processing, zero-valent metallic iron (one of the reaction educts) into its three-valent (ferric) state present in the reaction product. To the best of our knowledge, a mechanochemical reaction leading to such a large extent of oxidation of iron species (Fe^0^ → Fe^3+^) has not yet been reported. Note that the Fe^0^ → Fe^2+^ transformation path has been observed to occur, for example, during the single-step mechanosynthesis of nanostructured Fe^2+^-containing α-Fe_2_SiO_4_ and Fe_2_GeO_4_, starting from the mixture of metallic Fe^0^ and corresponding precursors (Šepelák et al., 2012a; Šepelák et al., 2012b), and the mechanochemical reduction of metal sulphides to prepare nanocrystalline metals (Godočíková et al., 2004). On the other hand, the mechanochemical reduction processes leading to the Fe^3+^ → Fe^2+^ → Fe^0^ transformations have been reported for spinel ferrites *M*Fe_2_O_4_ (*M* = Mg, Ni) (Menzel et al., 2001; Šepelák et al., 2002). The present work gives evidence of the fact that the unique mechanically triggered chemical reaction leading to the extensive oxidation of the Fe^0^ precursor is feasible. The evolution of the Sr_2_FeMoO_6_ phase during the mechanosynthesis as well as the far-from-equilibrium structural state of the reaction product are systematically monitored with X-ray diffraction (XRD) and a variety of comprehensive spectroscopic techniques including Raman spectroscopy, ^57^Fe Mössbauer spectroscopy, and X-ray photoelectron spectroscopy (XPS).

**FIGURE 1:**
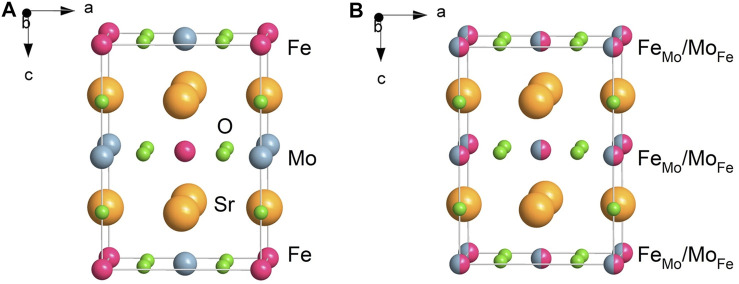
Schematic presentation of the tetragonal structure of **(A)** the *fully ordered* and **(B)** the *fully anti-site disordered* double perovskite Sr_2_FeMoO_6_. In the fully ordered Sr_2_FeMoO_6_, the Fe and Mo cations alternate regularly along the *c*-axis of the tetragonal crystal lattice. In the fully anti-site disordered Sr_2_FeMoO_6_, the cations are randomly distributed over the sites of octahedral coordination provided by the double perovskite structure. The crystal chemical formula of the fully disordered Sr_2_FeMoO_6_, emphasizing the cation site occupancy at the atomic level, may be written as Sr_2_[Fe_Fe_]_0.5_[Fe_Mo_]_0.5_[Mo_Mo_]_0.5_[Mo_Fe_]_0.5_O_6_, where [Fe_Fe_], [Mo_Mo_] and [Fe_Mo_], [Mo_Fe_] denote the cations on the “right” octahedral sites (Fe cation on Fe-site and Mo cation on Mo-site) and anti-sites (Fe cation on Mo-site and Mo cation on Fe-site), respectively.

A considerable interest in Sr_2_FeMoO_6_, from both fundamental and practical points of view, is prompted by the discovery of its room-temperature low-field magnetoresistance and half-metallicity (Kobayashi et al., 1998). Such properties predestinate this material for a variety of possible technological applications in the field of spintronics and solid oxide fuel cells (Kalanda et al., 2018; Huan et al., 2017; Skutina et al., 2021; Xi et al., 2021). Sr_2_FeMoO_6_ is an important member of the family of double perovskites with the general formula *A*_2_ *BB′*O_6_ (*A* is a divalent alkaline earth cation; *B* and *B′* are transition metal cations). It adopts the tetragonal crystal structure with space group *I*4/*m* (Nakamura and Oikawa, 2003), in which the twelve-fold coordinated Sr^2+^ cations reside in the cuboctahedral cavities created by the corner-sharing octahedra, while the octahedral sites are occupied by Fe and Mo ions. Despite its deceptively simple structure, Sr_2_FeMoO_6_ exhibits disordering phenomena involving the alternating positions of Fe and Mo atoms. In the fully ordered Sr_2_FeMoO_6_ double perovskite, Fe and Mo atoms alternate regularly along the *c*-axis of the tetragonal crystal lattice. Intervening O^2−^ ion bridges every Fe and Mo atom pair, thus forming the sequence of regularly alternating FeO_6_ and MoO_6_ octahedra along the *c*-axis (Figure 1). The cation disorder in Sr_2_FeMoO_6_, in which some Fe and Mo cations interchange their crystallographic positions, is referred to as anti-site disorder or mis-site disorder. The anti-site disordered regions (sometimes called “clusters” or “patches” (Menéndez et al., 2004)) can be easily visualized as islands consisting of oxygen octahedra whose centers are all occupied by Fe atoms or all by Mo atoms. The crystal chemical formula emphasizing the cation site occupancy in Sr_2_FeMoO_6_ may be written as Sr_2_[Fe_Fe_]_1-ASD_[Fe_Mo_]_ASD_[Mo_Mo_]_1-ASD_[Mo_Fe_]_ASD_O_6_, where [Fe_Fe_], [Mo_Mo_] and [Fe_Mo_], [Mo_Fe_] denote the cations on the “right” octahedral sites (Fe cation on Fe-site and Mo cation on Mo-site) and anti-sites (Fe cation on Mo-site and Mo cation on Fe-site), respectively. The symbol *ASD* represents the so-called degree of anti-site disorder defined as the fraction of Fe-sites occupied by Mo ions and *vice versa*. Theoretically, *ASD* takes a value from 0 to 0.5, i.e., 0 ≤ *ASD* ≤ 0.5. The value of *ASD* in Sr_2_FeMoO_6_ crucially depends on the conditions of its synthesis. For example, the lowest value (*ASD* = 0.025) has been found to be at an optimum synthesis temperature of about 1423 K for ceramics long-term annealed (up to 150 h) under oxygen-deficient conditions (Shimada et al., 2003; Huang et al., 2004). On the other hand, the considerably high values of *ASD* ranging from 0.345 to 0.45 have been reported for Sr_2_FeMoO_6_ prepared by melt-quenching method (Sarma et al., 2000a; Kobayashi et al., 2007). Here, we were able to mechanosynthesize the nanostructured and fully anti-site disordered Sr_2_FeMoO_6_ with a record-high value of *ASD* = 0.5. It should be emphasized that this value, derived independently from the present experimental XRD, Mössbauer, and magnetization data, is, to the best of our knowledge, the highest value so far reported for the Sr_2_FeMoO_6_ phase.

Anti-site defects are recognized to drastically influence the magnetism of Sr_2_FeMoO_6_ (Reyes et al., 2016; Kalanda et al., 2020). The present study demonstrates that an extraordinarily high *ASD* in the as-prepared double perovskite leads to the deterioration of its magnetic properties. The mechanosynthesized Sr_2_FeMoO_6_ exhibits superparamagnetism at room temperature arising from its nanoscale nature evidenced by high-resolution transmission electron microscopy (HRTEM).

## Materials and Methods

For the mechanosynthesis of Sr_2_FeMoO_6_, the reactants SrO (Alfa Aesar), α-Fe (Alfa Aesar) and MoO_3_ (Sigma-Aldrich) with the initial average particle sizes of 0.5, 0.7 and 0.3 μm, respectively, were mixed in a molar ratio of 2:1:1. The 2 SrO + α-Fe + MoO_3_ mixtures were milled for various times *t*_M_ (up to 240 min) in a planetary ball mill Fritsch Pulverisette 7 Premium Line (Fritsch, Germany) at room temperature. The milling chamber (80 cm^3^ in volume) and 15 balls (10 mm in diameter), both made of tungsten carbide, were used. Milling experiments were performed in air at 500 rpm.

The XRD patterns were measured using a D8 Advance diffractometer (Bruker, Germany), operating in Bragg-Brentano configuration and using Cu Kα radiation. The XRD scans were collected from 10° to 70° (2 Theta), using a step of 0.03° and a data collection time of 12 s. The Powder Diffraction File (PDF) database (ICDD, Newtown Square, PA, United States) was utilized for phase identification. The tetragonal double perovskite structure of Sr_2_FeMoO_6_ was visualized using the Diamond program (Putz and Brandenburg, 2019).

Raman spectra were recorded using a micro-Raman multichannel spectrometer LabRam300 Infinity (Horiba Jobin Yvon, Japan) with the excitation wavelength of 532 nm (Nd:YAG laser). The Raman shift was calibrated using the Raman peak of silica wafer positioned at 520.7 cm^−1^. The acquisition time and the number of scans were set to 10 and 7 s, respectively.

^57^Fe Mössbauer spectra were taken in transmission geometry at room temperature (293 K) and 13 K. ^57^Co in Rh matrix was used as the γ-ray source. Mössbauer data were fitted using the *WinNormos* software package (Wissel, Germany). The derived isomer shifts (IS) are given relative to IS of α-Fe at room temperature. The degree of anti-site disorder was calculated according to formula *ASD* = *I*[Fe_Mo_]/2(*I*[Fe_Fe_] + *I*[Fe_Mo_]), where *I*[Fe_Mo_] and *I*[Fe_Fe_] are the relative intensities of Mössbauer subspectra corresponding to Fe ions on the Mo-sites and Fe-sites, respectively.

XPS measurements were performed using an XPS instrument SPECS (Specs, Germany) equipped with PHOIBOS 100 SCD and non-monochromatic X-ray source. The spectra were acquired at a basic pressure of 2 × 10^−8^ mbar with Mg Kα excitation at 10 kV. The spectrometer was calibrated against silver (Ag 3*d*). The data were analyzed using the *SpecsLab2 CasaXPS* software (Casa Software, United Kingdom).

HRTEM observations were performed employing a JEOL 2100F UHR microscope operated at 200 kV. High-resolution images were taken in a bright-field imaging mode. Prior to HRTEM investigations, powders were dispersed in ethanol and ultrasonicated for 10 min. Subsequently, a droplet of the water-diluted colloidal suspension was deposited on the copper-supported carbon grid. Finally, samples were stored in a vacuum chamber to evaporate residual ethanol.

Magnetic measurements were performed using a superconducting quantum interference device (SQUID) magnetometer (Quantum Design MPMS-5S). The powdered samples were filled in a small gelatin capsule, whose diamagnetic moment was subtracted from the measured magnetization values. Magnetic hysteresis loops were recorded at 10 and 300 K in external magnetic fields from 0 to ±5 T. The temperature-dependent magnetic susceptibility was measured from 5 to 300 K in a magnetic field of 0.01 T.

## Results and Discussion

**FIGURE 2:**
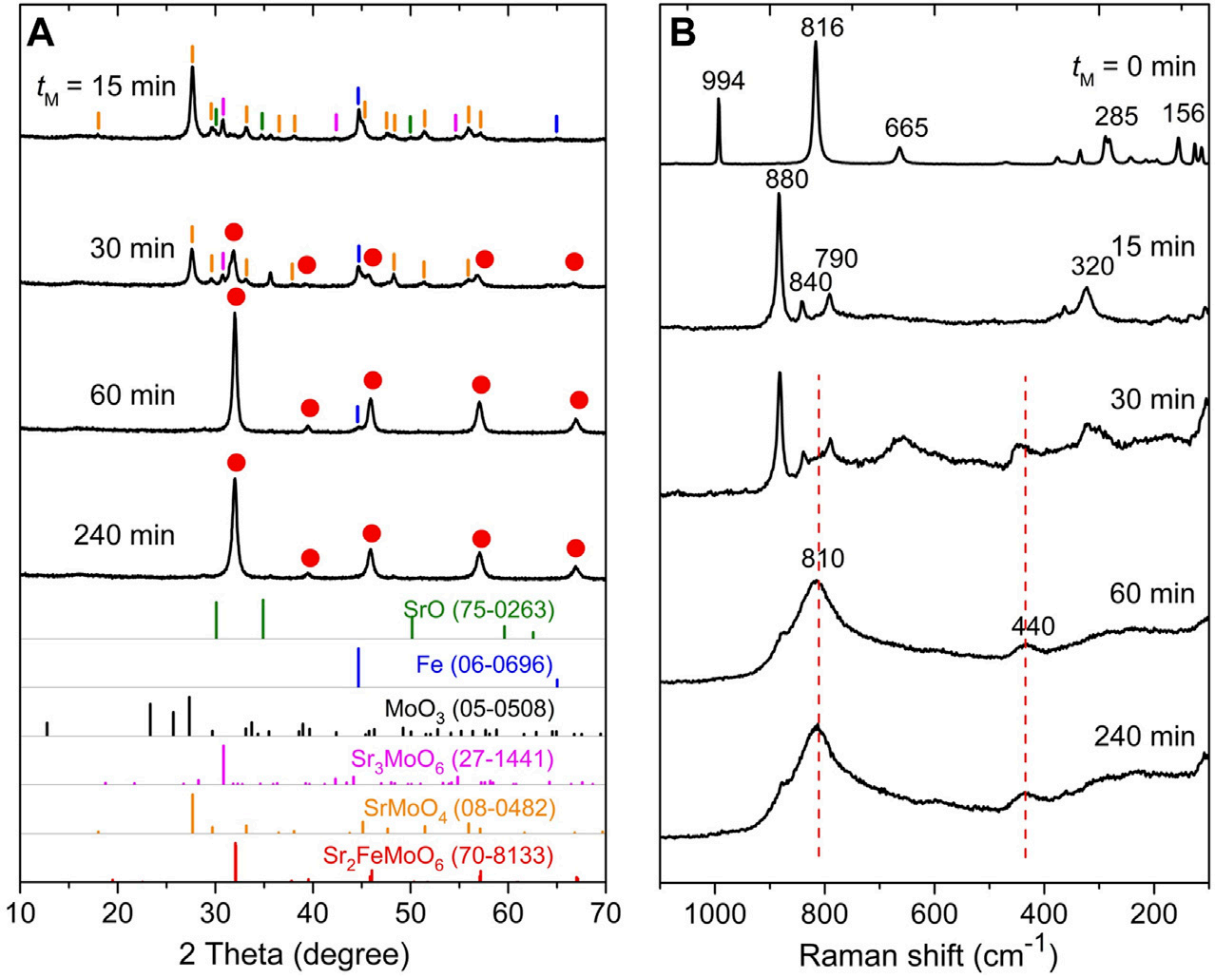
**(A)** XRD patterns and **(B)** Raman spectra of the 2 SrO + α-Fe + MoO_3_ mixture milled for various times (*t*_M_) up to 240 min in air. The mechanochemical reaction 2 SrO + α-Fe + MoO_3_ + ½ O_2_ →
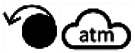
→ Sr_2_FeMoO_6_ proceeds through the formation of intermediate phases SrMoO_4_ and Sr_3_MoO_6_. The theoretical Bragg peak positions with the ICDD PDF numbers corresponding to the reaction precursors, intermediate phases and the product phase are presented at the bottom of panel 2A. The positions of Raman peaks associated with the formed Sr_2_FeMoO_6_ phase are indicated in Panel 2B by the dashed lines.

The evolution of the 2 SrO + α-Fe + MoO_3_ mixture milled in air was followed by XRD. Figure 2A shows XRD patterns of the mixture milled for various times. The XRD pattern of the starting powder (not shown) is characterized by sharp diffraction peaks corresponding to the solid reactants SrO (ICDD PDF 75-0263), α-Fe (06-0696) and MoO_3_ (05-0508). During the early stages of milling (for *t*_M_ ≤ 15 min), XRD reveals a decrease in the intensity and an associated broadening of the Bragg peaks of the SrO and α-Fe educts, the complete disappearance of diffraction peaks assigned to the binary MoO_3_ precursor, and the simultaneous formation of the intermediate ternary phase SrMoO_4_ (08-0482); see Figure 2A (top). Note that a tiny fraction of Sr_3_MoO_6_ (27-1441) has also been detected in the sample milled up to 30 min. In analogy with our previous work on the mechanosynthesis of oxymolybdates (Tóthová et al., 2019) it can be assumed that a rapid formation of the ternary molybdates is accelerated by the emergence of the structurally disordered (or even amorphous) and, consequently, very reactive MoO_3_ phase induced by mechanical action already in the early stages of milling process. For milling times *t*_M_ ≥ 30 min, qualitative changes are observed in the XRD patterns of the milled samples; clear evidence is observed of new diffraction features that correspond to the complete disappearance of the intermediates and the simultaneous formation of the Sr_2_FeMoO_6_ quaternary phase. In the XRD pattern of the mixture milled for 240 min (a product of the mechanochemical reaction), all diffraction peaks detected above the background are due to the tetragonal Sr_2_FeMoO_6_ phase (70-8133); see Figure 2A (bottom). Rietveld analysis of XRD data revealed the lattice parameters (*a* = 5.5856(5) Å, *c* = 7.8970(140) Å) and the random distribution of Fe and Mo cations (*ASD* = 0.5) in the mechanosynthesized Sr_2_FeMoO_6_. The latter will be discussed in detail concurrently with the analysis of the low-temperature ^57^Fe Mössbauer spectroscopic data and SQUID magnetization data (see below).

To provide insight into the formation of intermediates in greater detail, the mechanochemical reaction 2 SrO + α-Fe + MoO_3_ + ½ O_2_ →
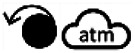
→ Sr_2_FeMoO_6_ was also followed by Raman spectroscopy. Figure 2B compares Raman spectra of the 2 SrO + α-Fe + MoO_3_ mixtures milled for various *t*_M_. The spectrum of the starting mixture (*t*_M_ = 0 min) shows all Raman peaks typical for MoO_3_ reactant. Note that the first-order Raman bands are neither expected for *bcc* structure of α-Fe nor for SrO being two remaining solid reactants (Mon, 1972). The strongest Raman peaks at 816 cm^−1^ (with symmetries *A*_g_, *B*_1g_) and 994 cm^−1^ (*A*_g_, *B*_1g_) evolve from the asymmetric and symmetric stretching of the terminal oxygen atoms in MoO_3_, whereas the band at 665 cm^−1^ (*B*_2g_, *B*_3g_) appears from the asymmetric stretching of the Mo–O–Mo bridge along the *c*-axis (Mestl et al., 1994). The lower wavenumber bands at 281 and 289 cm^−1^ (*B*_2g_, *B*_3g_) are ascribed to the wagging modes of the terminal oxygen atoms, in addition to the 156 cm^−1^ band (*A*_g_, *B*_1g_) that originates from the translation of rigid chains. An interesting observation is that already after 15 min of high-energy milling, all the Raman peaks assigned to the MoO_3_ precursor disappear, and new bands at 880, 840, 790, and 320 cm^−1^ evolve (Figure 2B). These bands are attributed to the formed SrMoO_4_ intermediate phase (Sujatha et al., 2019). This finding supports and complements the above given XRD results, which reveal the complete and rapid reaction of MoO_3_ and SrO precursors yielding the intermediate ternary molybdate SrMoO_4_ already in the early stages of milling process. Further milling (*t*_M_ > 15 min) provokes significant changes in the Raman spectra; the most substantial feature is the appearance of a broad and intense complex band peaking at about 810 cm^−1^ (ranging from about 850 to 770 cm^−1^) that, in addition to the 440 cm^−1^ weaker band, is characteristic for Sr_2_FeMoO_6_ (Son et al., 2001; Navarro et al., 2003). The broad shape of these Raman peaks reflects the structurally disordered nature of the final reaction product. Taking into account the results of the present XRD and Raman spectroscopic investigations, the overall reaction path leading to Sr_2_FeMoO_6_ and involving the formation of the SrMoO_4_ intermediate can be expressed by the following sequence of two mechanochemical reactions: 2 SrO + α-Fe^0^ + Mo^6+^O_3_ + ½ O_2_ →
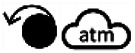
→ SrO + α-Fe^0^ + SrMo^6+^O_4_ + ½ O_2_ →
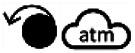
→ Sr_2_Fe^3+^Mo^5+^O_6_. The first reaction represents the *mechanochemical synthesis* of SrMo^6+^O_4_, which is not accompanied by the valence change of the constituent cations, whereas the subsequent *mechanochemical redox reaction* includes the reduction of Mo^6+^ cations and the oxidation of Fe^0^ atoms. Note that a small amount of the SrMoO_4_ intermediate remains unreacted in the sample milled up to 240 min (see a tiny Raman peak at 880 cm^−1^ at the bottom of Figure 2B).

**FIGURE 3:**
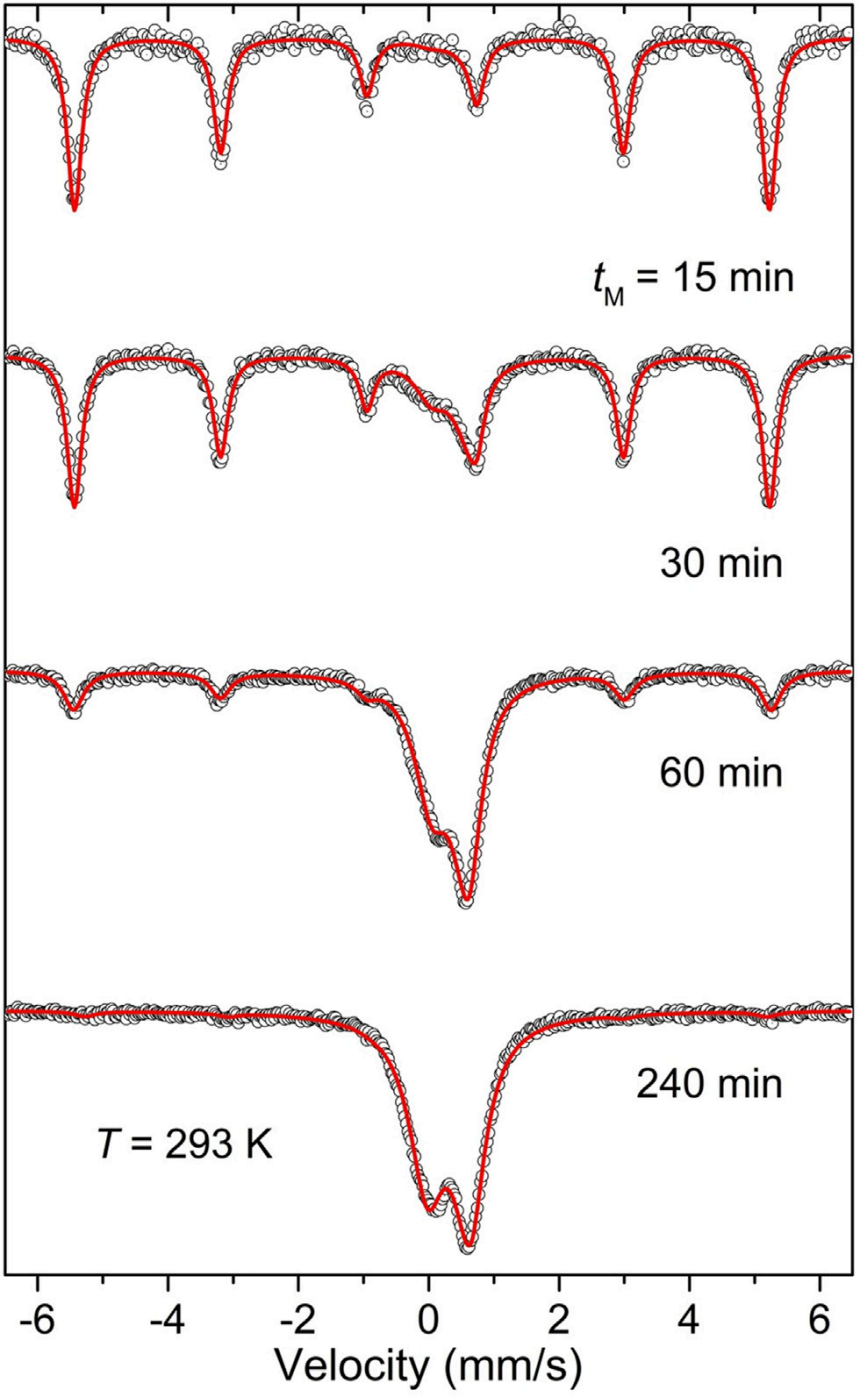
Room-temperature ^57^Fe Mössbauer spectra of the 2 SrO + α-Fe + MoO_3_ mixture milled for various times (*t*_M_) up to 240 min.

To determine the mechanically induced phase evolution of the 2 SrO + α-Fe + MoO_3_ mixture from the point of view of the α-Fe precursor as an oxygen getter and to provide complementary insight into the short-range (local) structure of the formed Sr_2_FeMoO_6_ phase, the mechanochemical reaction was also followed by ^57^Fe Mössbauer spectroscopy. This nuclear spectroscopic method has been proven to be well suited for the investigation of the charge state, the local coordination, and the magnetic state of iron ions in mechanosynthesized materials (Šepelák and Becker, 2000; Šepelák et al., 2006; Šepelák et al., 2007; Da Silva et al., 2011; Harris and Šepelák, 2018; Tóthová et al., 2018; Skurikhina et al., 2020). The room-temperature ^57^Fe Mössbauer spectra illustrating the mechanically induced evolution of the 2 SrO + α-Fe + MoO_3_ mixture are presented in Figure 3. As can be seen, the spectrum of the starting mixture shows a sextet with isomer shift IS = 0.02(1) mm/s and magnetic hyperfine field *B*_hf_ = 33.1(1) T corresponding to *bcc* metallic Fe in ferromagnetic state. With increasing *t*_M_, the sextet becomes asymmetric toward the inside of each line, slowly collapses, and is gradually replaced by a central asymmetric doublet. In the spectrum of the sample milled for 240 min (see Figure 3, bottom), the sextet corresponding to α-Fe is not visible anymore and the central doublet becomes the only spectral component. Considering the relatively high ferrimagnetic-to-paramagnetic transition temperature of bulk (microcrystalline) Sr_2_FeMoO_6_ (*T*_c_ = 415 K (Kobayashi et al., 1998)), the presence of the doublet in the room-temperature ^57^Fe Mössbauer spectrum of the sample milled for 240 min seems to be an unexpected finding. It can be understood to arise mostly from Fe cations in mechanosynthesized Sr_2_FeMoO_6_ nanoparticles exhibiting *superparamagnetism* (see below). Note that the latter arises if particle sizes are so small that thermally induced energy fluctuations (∼*k*_B_ *T*, where *k*_B_ is the Boltzmann constant and *T* is temperature) overcome the magnetic anisotropy energy (∼*KV*, where *K* is the anisotropy energy constant and *V* is the particle volume) and change the direction of the magnetization of a particle from one easy axis to another (Long, 1987). Similar magnetic relaxation phenomena and the complete absence of the magnetic hyperfine splitting at room temperature have been observed in other Fe-containing ultrafine-particle magnetics (e.g., ferrites) prepared *via* mechanosynthesis (Šepelák et al., 2006; Šepelák et al., 2007; Da Silva et al., 2011).

**FIGURE 4:**
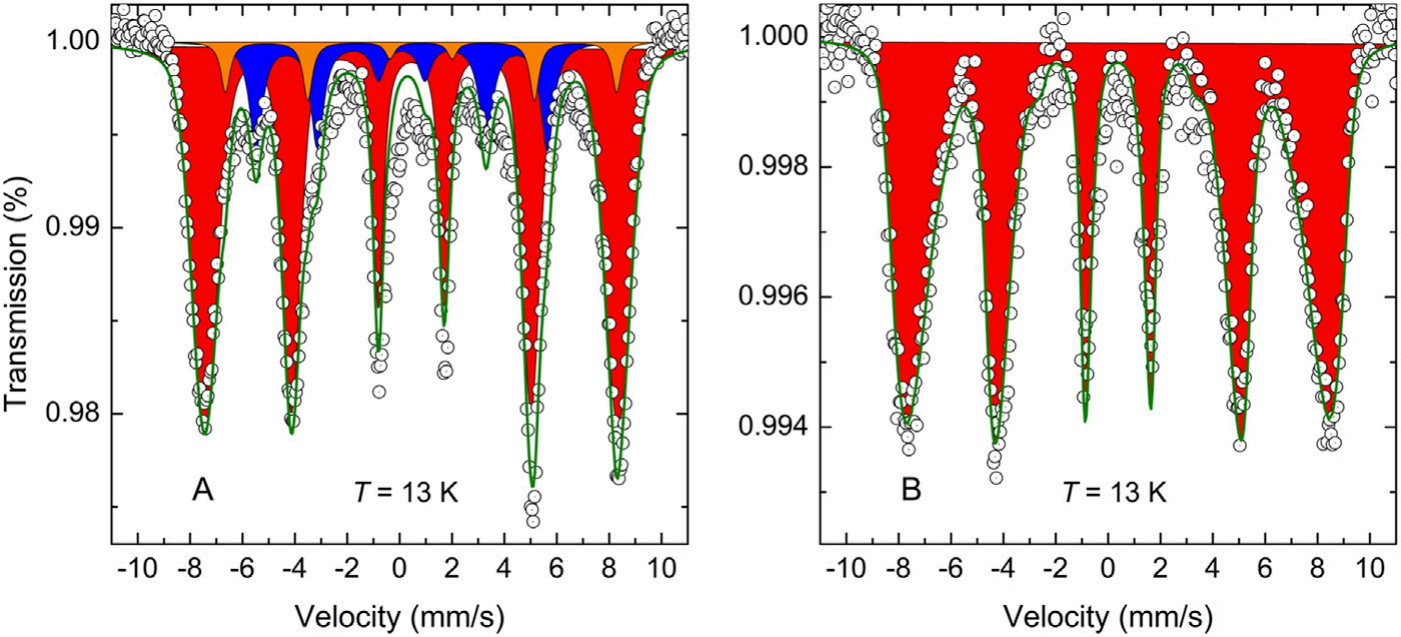
The low-temperature (13 K) ^57^Fe Mössbauer spectra of the 2 SrO + α-Fe + MoO_3_ mixture milled for 240 min **(A)** before and **(B)** after thermal treatment at 923 K for 30 min in air. The spectra correspond to **(A)** the partly synthesized Sr_2_FeMoO_6_ and **(B)** the completely synthesized Sr_2_FeMoO_6_. The orange and red subspectra are associated with Fe cations in the *ordered* and *anti-site disordered regions* of Sr_2_FeMoO_6_ double perovskite, respectively. The blue subspecrum corresponds to α-Fe.

Since the relaxation time characterizing the superparamagnetic phenomenon is also a function of temperature (*τ* ∼ exp(*KV*/*k*_B_ *T*)) (Néel, 1949), the influence of superparamagnetic relaxation can be counteracted by reducing the sample temperature. Figure 4A presents the low-temperature (13 K) ^57^Fe Mössbauer spectrum of the 2 SrO + α-Fe + MoO_3_ mixture milled for 240 min. It is clearly visible that thermal fluctuations are suppressed at 13 K, and the Mössbauer spectrum of the sample consists of the superposition of three magnetic hyperfine splitting components (sextets). The hyperfine parameters resulting from the least-squares fitting of the spectrum are presented in Table 1. The first minor sextet (orange subspectrum with the relative intensity *I*[Fe_Fe_] ∼ 5.9%) is assigned to the Fe cations in the *ordered regions (clusters)* of the mechanosynthesized Sr_2_FeMoO_6_ double perovskite with the regularly alternating corner-sharing FeO_6_ and MoO_6_ octahedra along the *c*-axis of its tetragonal crystal lattice. The value IS = 0.92(6) mm/s for this sextet corresponds to the state with a mixed valence of iron cations Fe^2+^/Fe^3+^ (between Fe^2+^ and Fe^3+^; sometimes formally denoted as Fe^2.5+^) (Greneche et al., 2001; Menéndez et al., 2004). This degenerate nature of the fundamental state of iron cations in Sr_2_FeMoO_6_ is the result of the existing equilibrium Fe^3+^ + Mo^5+^ ↔ Fe^2+^ + Mo^6+^, in which the itinerant down-spin electron is shared by both types of atoms (Sarma et al., 2000b; Lindén et al., 2000).

**TABLE 1:** Parameters obtained by fitting ^57^Fe Mössbauer spectra taken at 13 and 293 K for the 2 SrO + α-Fe + MoO_3_ mixture milled for 240 min before and after its thermal treatment. IS is the isomer shift; QS is the quadrupole splitting; *B*_hf_ is the magnetic hyperfine field; *I* is the relative intensity of the spectral component. Symbols [Fe_Fe_] and [Fe_Mo_] denote iron cations in octahedral coordination of oxygen anions in the *ordered* and *anti-site disordered regions* of Sr_2_FeMoO_6_, respectively.

*T* (K)	Material	Spectral component assigned to	IS (mm/s)	QS (mm/s)	*B*_hf_ (T)	*I* (%)
13	Product of the 2 SrO + α-Fe + MoO_3_ mixture milled for 240 min	[Fe_Fe_]	0.92(6)	—	46.4(1)	5.9(1)
		[Fe_Mo_]	0.55(1)	—	49.0(3)	84.1(6)
		α-Fe	0.18(4)	—	34.5(4)	10.0(2)
13	Product of the 2 SrO + α-Fe + MoO_3_ mixture milled for 240 min followed by thermal treatment	[Fe_Mo_]	0.48(2)	—	48.8(2)	100
293	Product of the 2 SrO + α-Fe + MoO_3_ mixture milled for 240 min followed by thermal treatment	[Fe_Mo_]	0.35(5)	0.92(1)	—	100

The major component in the spectrum shown in Figure 4A (red subspectrum with the relative intensity *I*[Fe_Mo_] ∼ 84.1%) is ascribed to the high-spin Fe^3+^ cations in octahedral coordination of oxygen anions in the *anti-site disordered regions* of the mechanosynthesized Sr_2_FeMoO_6_, where Mo-sites are occupied by Fe^3+^ cations and *vice versa*. From the relative intensities *I*[Fe_Mo_] and *I*[Fe_Fe_], the degree of anti-site disorder in the mechanosynthesized Sr_2_FeMoO_6_ is estimated to be *ASD* ∼ 0.467. Thus, the mechanosynthesized Sr_2_FeMoO_6_ can be considered as a nonequilibrium, highly disordered double perovskite possessing the almost random distribution of the Fe and Mo cations over the sites of octahedral coordination provided by the double perovskite structure. It should be noted that the iron nuclei located on the anti-sites ([Fe_Mo_]) experience the most probable local magnetic hyperfine field *B*_hf_ = 49.0(3) T with a relatively broad field distribution of about 10 T (from about 45 to 55 T). This fact is a strong indication of both, the presence of a wide variation in the local chemical environment around Fe^3+^ cations from site to site and the presence of a strongly distorted geometry of FeO_6_ octahedra in the anti-site disordered clusters of the mechanosynthesized material. This is in contrast to the iron cations located on the “right” octahedral sites ([Fe_Fe_]), where the magnetic hyperfine field with the lower magnitude (*B*_hf_ = 46.4(1) T) and relatively narrow distribution of about 5 T is observed.

The Mössbauer active ^57^Fe nuclei provide a very sensitive probe for the estimation of the yield of a mechanochemical formation reaction of Fe-containing compounds (Šepelák et al., 2006; Da Silva et al., 2011). The presence of the third sextet, that is assigned to metallic α-Fe nanoparticles (blue subspectrum with the relative intensity *I*_α-Fe_ ∼ 10% in Figure 4A), indicates that the present mechanochemical reaction is not completed after 240 min; its degree of conversion is about 90%. Note that this sextet is not present in the corresponding room-temperature Mössbauer spectrum (see Figure 3, bottom), confirming the superparamagnetic state of α-Fe nanoparticles in the mixture of the milled reactants. Taking into account that the present heterogeneous mechanochemical reaction involving solid precursors and the gaseous phase (oxygen) proceeds inside the closed milling chamber, it can be assumed that the further progress of the mechanosynthesis of Sr_2_FeMoO_6_ was hampered by the shortage of oxygen inside the chamber, and the reaction stopped after the full consumption of oxygen. However, the subsequent thermal treatment of the sample at a fairly moderate temperature of 923 K for 30 min in air leads to the further reaction of remaining α-Fe nanoparticles in the reaction mixture, and results in the full (100%) conversion of the reactants into the double perovskite Sr_2_FeMoO_6_ phase. This is documented in Figure 4B, where the low-temperature ^57^Fe Mössbauer spectrum of the 2 SrO + α-Fe + MoO_3_ mixture milled for 240 min taken after its thermal treatment is shown. The hyperfine parameters of the as-prepared Sr_2_FeMoO_6_ are listed in Table 1. It should be emphasized that the ^57^Fe Mössbauer spectrum of the completely synthesized Sr_2_FeMoO_6_ consists of only one spectral component corresponding to the *fully anti-site disordered* Sr_2_FeMoO_6_ double perovskite with *ASD* = 0.5. This extraordinarily high value is, to the best of our knowledge, the highest value so far reported for the anti-site disordered Sr_2_FeMoO_6_ phase.

An interesting observation is that the moderate thermal tretament leads to the transformation of the small amount (∼5.9 wt.%) of the ordered Sr_2_FeMoO_6_ clusters into the anti-site disordered state. This unusual structural order-disorder transformation upon heating can be caused by the progression of concurrent reaction of remaining Fe^0^ (∼10 wt.%) in the reaction mixture. It seems that tiny metallic iron in superparamagnetic state with a large reaction surface and a high oxidation affinity plays an important role in the present mechanosynthesis of Sr_2_FeMoO_6_. Its mechanically induced steady presence in the reaction mixture of educts initiates/promotes the redox Fe^0^ → Fe^3+^ and Mo^6+^ → Mo^5+^ reactions.

**FIGURE 5:**
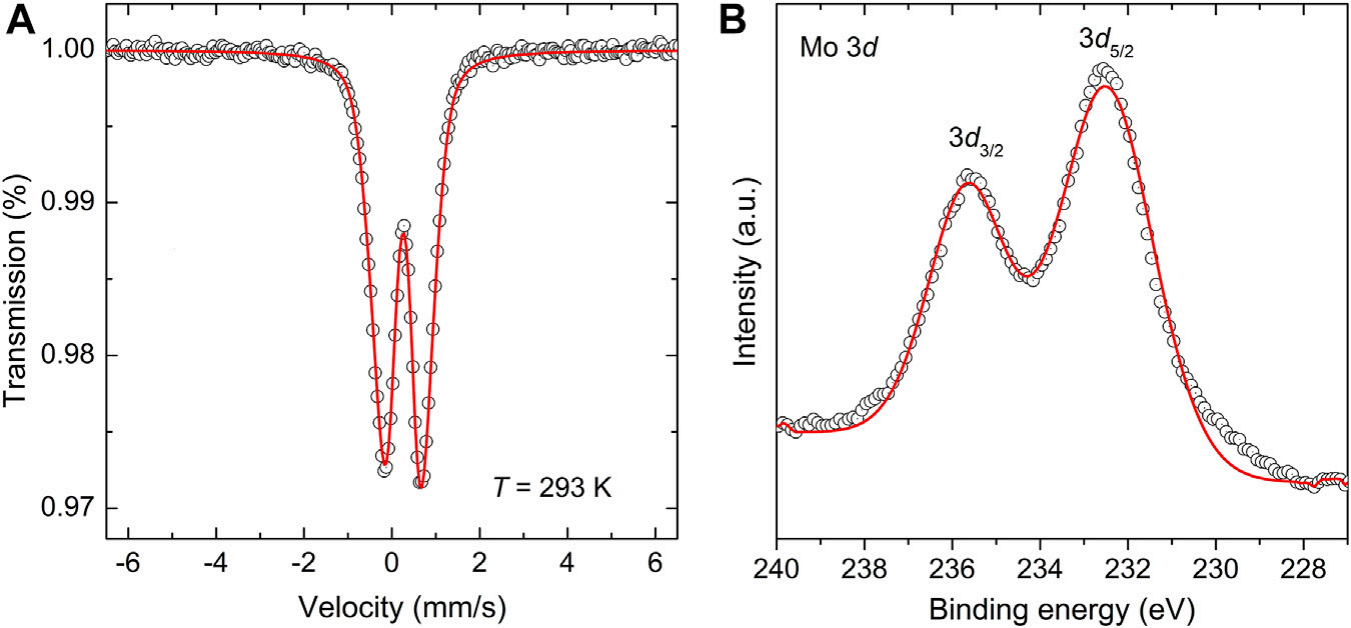
**(A)** The room-temperature ^57^Fe Mössbauer spectrum and **(B)** the Mo *3d* core level XPS spectrum of the as-prepared Sr_2_FeMoO_6_.

To verify the valence state of the Fe and Mo ions in the as-prepared fully anti-site disordered double perovskite, information on its short-range structure, provided by the ^57^Fe Mössbauer spectroscopic technique, is complemented by an investigation of the electronic state of the constituent cations by means of X-ray photoelectron spectroscopy. Figure 5 displays both the room-temperature ^57^Fe Mössbauer and XPS spectra of the material. The ^57^Fe Mössbauer spectrum (Figure 5A) consists of the symmetric doublet that is well fitted by only one spectral component with isomer shift IS = 0.35(5) mm/s and quadrupole splitting QS = 0.92(1) mm/s. The latter values of hyperfine parameters are characteristic of Fe^3+^ cations in octahedral coordination of oxygen anions (Menil, 1985). The presence of the doublet also reveals that, even after its moderate thermal treatment, the as-prepared fully anti-site disordered Sr_2_FeMoO_6_ double perovskite exhibits superparamagnetism at room temperature (see doublets in Figure 2 (bottom) and Figure 5A). This demonstrates a fairly good stability of the superparamagnetic material at moderate temperatures, and simultaneously indicates that the thermal treatment applied does not bring about a substantial growth of Sr_2_FeMoO_6_ nanoparticles (see also the detailed HRTEM analysis below). It is also worth to mention that the value of quadrupole splitting (QS ∼ 0.9 mm/s) for the as-prepared double perovskite is significantly larger than that observed for other superparamagnetic fine Sr_2_FeMoO_6_ nanoparticles (Yarmolich et al., 2016; Suominen et al., 2007). This variation is associated with the presence of strongly deformed FeO_6_ octahedra in the mechanosynthesized perovskite, as it is derived from the low-temperature ^57^Fe Mössbauer spectra presented in Figure 4. Note that the broadly distorted geometry of the constituent structural units (building blocks) is typical for nanosized particles prepared by mechanochemical routes, and it has been evidenced by nuclear spectroscopic methods in numerous far-from-equilibrium complex oxides (Šepelák et al., 2009; Šepelák et al., 2013; Da Silva et al., 2021). This is also in line with previous work on Sr_2_FeMoO_6_, where an important crystallographic distortion around Fe^3+^ cations is postulated due to highly unspherical electric fields generated by anti-side disorder (Greneche et al., 2001) or, alternatively, due to the existence of Mo vacancies (Chmaissem et al., 2000).

The Mo *3d* core level XPS spectrum of the as-prepared Sr_2_FeMoO_6_ is displayed in Figure 5B. In the XPS spectrum of the initial 2 SrO + Fe + MoO_3_ mixture (the spectrum not shown), the Mo *3d* core electron level appears at the binding energies of 232.1 and 235.2 eV for 3*d*_5/2_ and 3*d*_3/2_ electronic states, respectively. These values are typical for the Mo^6+^ valence state in the MoO_3_ educt (Maiti et al., 2019). With increasing milling time, i.e., with increasing amount of the Sr_2_FeMoO_6_ phase in the reaction mixture, the 3*d*_5/2_ and 3*d*_3/2_ electron sublevels shift to sligtly higher binding energies. For the mechanosynthesized Sr_2_FeMoO_6_, the structureless 3*d*_5/2_ and 3*d*_3/2_ peaks are observed at 232.6 and 235.7 eV, respectively, (see Figure 5B). Using the literature values for different Mo oxides (Sarma et al., 2000b; Jalili et al., 2009), the observed Mo 3*d*_5/2_ and Mo 3*d*_3/2_ features are ascribed to arise solely from the Mo^5+^ oxidation state. Note that the similar values of binding energies (232.2 and 235.3 eV) have been reported for the Mo^5+^ state in nanoscale thin films of Sr_2_FeMoO_6_ (Jalili et al., 2009). Thus, it is clear from the analyses given hitherto that the present mechanochemical redox reaction can be written as: 2 SrO + α-Fe^0^ + Mo^6+^O_3_ + ½ O_2_ →
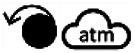
→ Sr_2_Fe^3+^Mo^5+^O_6_, where Mo^6+^ is reduced to Mo^5+^ and Fe^0^ is oxidized to Fe^3+^. To the best of our knowledge, the one-step mechanochemical synthesis accompanied by such a large extent of oxidation (Fe^0^ → Fe^3+^) has not yet been reported.

**FIGURE 6:**
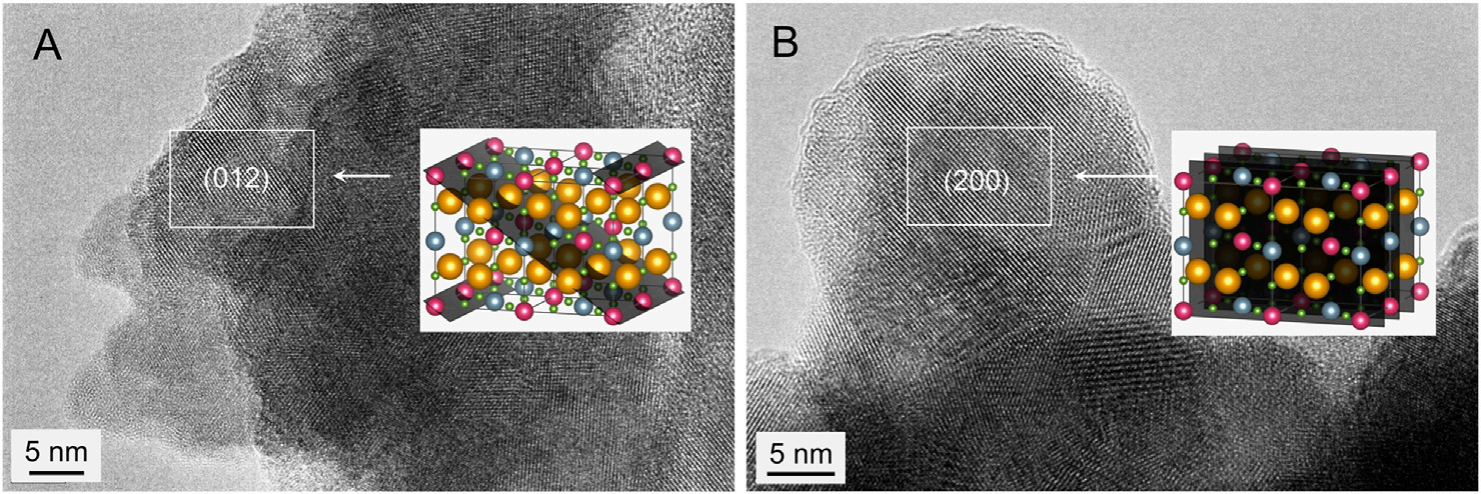
HRTEM images of **(A)** the partly synthesized Sr_2_FeMoO_6_ and **(B)** the completely synthesized Sr_2_FeMoO_6_. The lattice fringes correspond to the crystallographic planes **(A)** (012) with interplanar distance *d*_(012)_ = 0.36 nm and **(B)** (200) with *d*_(200)_ = 0.28 nm of the Sr_2_FeMoO_6_ phase (ICDD PDF 70-8133).

Taking into account that superparamagnetism, evidenced for the as-prepared materials by means of ^57^Fe Mössbauer spectroscopy (see above), is the particle size (the particle volume) dependent phenomenon (Néel, 1949), HRTEM investigations of both the partly synthesized Sr_2_FeMoO_6_ (the 2 SrO + Fe + MoO_3_ mixture milled for 240 min) and the completely synthesized Sr_2_FeMoO_6_ (the mixture milled for 240 min followed by its thermal treatment at 923 K for 30 min) were performed to uncover their morphology. The representative HRTEM micrographs of the as-prepared materials are shown in Figure 6. It is revealed that both materials consist of agglomerated fully crystalline nanoparticles. The partly synthesized Sr_2_FeMoO_6_ is found to exhibit a particle size distribution ranging from about 5 to 12 nm (an average particle size is estimated to be 9(2) nm) with the irregular particle shape and random orientation, whereas the completely synthesized Sr_2_FeMoO_6_ possesses a broader particle size distribution (7–28 nm) with the average particle size of about 21(4) nm and the roughly spherical particle shape. The relatively broad distribution of particle sizes can be a result of the heterogeneous nucleation-and-growth processes (Da Silva et al., 2011) of the product phase in the course of the mechanically induced formation reaction. In both cases, the high-resolution TEM images show lattice fringes corresponding to the crystallographic planes with Miller indices (012) (interplanar distance *d*_(012)_ = 0.36 nm) (Figure 6A) and (200) (*d*_(200)_ = 0.28 nm) (Figure 6B) of the Sr_2_FeMoO_6_ phase (ICDD PDF 70-8133). The lattice fringes cross the whole interior of particles demonstrating their single-crystalline character. Thus, it is evident now that the applied moderate thermal treatment of the partly synthesized Sr_2_FeMoO_6_ nanoparticles does not bring about their substantial growth; in the thermally treated sample, Sr_2_FeMoO_6_ nanoparticles are still sufficiently small to exhibit superparamagnetic state at room-temperature (see Figure 5A).

**FIGURE 7:**
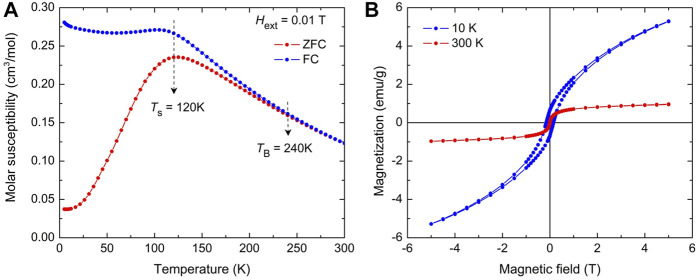
**(A)** Zero-field-cooled (ZFC) and field-cooled (FC) magnetization curves for the as-prepared Sr_2_FeMoO_6_ taken at external magnetic field *H*_ext_ = 0.01 T. *T*_s_ and *T*_B_ denote blocking temperatures of the smallest and the biggest Sr_2_FeMoO_6_ nanoparticles in the double perovskite particulate system, respectively. **(B)** Magnetization hysteresis loops for the as-prepared Sr_2_FeMoO_6_ measured at 10 K (blue line) and 300 K (red line).

The temperature-dependent magnetization measurements were performed to confirm the superparamagnetic state of the as-prepared nanostructured Sr_2_FeMoO_6_. Figure 7A shows the zero-field-cooled (ZFC) and field-cooled (FC) magnetization curves of the nanomaterial measured in the temperature range from 5 to 300 K in an external magnetic field of 0.01 T. Above 5 K, the increase with temperature of the ZFC curve is an indicator of the appearance of superparamagnetism in Sr_2_FeMoO_6_ nanoparticles, which give rise to a single maximum at about *T*_s_ = 120 K (Hansen and Mørup, 1999). The latter is a critical point (the so-called *blocking temperature*) corresponding to the blocking (freezing of the magnetic moments) of the smallest nanoparticles in the double perovskite particulate system. The point at which the ZFC and FC curves start to coincide (*T*_B_ ∼ 240 K) is associated with the blocking temperature of the biggest Sr_2_FeMoO_6_ nanoparticles. The relatively large difference between *T*_s_ and *T*_B_ (∼120 K) indicates that the as-prepared double perovskite possesses a relatively broad distribution of particle sizes. The latter finding confirms the results of HRTEM investigations presented above. The presence of a single maximum in the ZFC curve points to a single-stage process of the nanoparticle blocking (Yu et al., 1999), which reflects a single-modal particle size distribution in the nanostructured material.

There is a strong correlation between the octahedral cation anti-site disorder in Sr_2_FeMoO_6_ and its magnetism (Kobayashi et al., 1998; Ogale et al., 1999; Reyes et al., 2016). The magnetic ground state of the fully ordered Sr_2_FeMoO_6_ is usually considered as a ferrimagnetic collinear state with parallel magnetic moments of Fe^3+^ cations (*μ*_Fe_ = 5 *μ*_B_, *μ*_B_ is Bohr magneton; *μ*_B_ = 9.27408 × 10^−21^ emu), which are antiparallel to the moments of Mo^5+^ cations (*μ*_Mo_ = 1 *μ*_B_) (Saloaro et al., 2016). Taking into account the formula Sr_2_[Fe_Fe↑_]_1-ASD_[Fe_Mo↓_]_ASD_[Mo_Mo↓_]_1-ASD_[Mo_Fe↑_]_ASD_O_6_, which emphasizes the cation site occupancy and the spin alignment in the anti-site disordered Sr_2_FeMoO_6_, the *effective magnetic moment μ* per formula unit (f.u.) of the material may be written as:

$$\begin{array}{l} \mu \;=\;\mu_{\text{Fe}}\left(1-ASD\right)-\mu_{\text{Fe}}ASD-\mu_{\text{Mo}}\;\left(1-ASD\right)+\mu_{\text{Mo}}ASD=\mu_{\text{Fe}}\left(1-2ASD\right)-\mu_{\text{Mo}}\left(1-2ASD\right)=\left(\mu_{\text{Fe}}-\mu_{\text{Mo}}\right)\\ \left(1-2ASD\right)=4\mu_{\text{B}}\left(1-2ASD\right)=\left(4-8ASD\right)\mu_{\text{B}} \end{array}$$

Thus, this simplified approach taking into account the spin-only contribution of cations and omitting the itinerant character of spin-down electrons shows that *μ* linearly decreases with increasing *ASD*. The effective magnetic moment *μ* = 4 *μ*_B_/f.u. corresponds to the fully ordered double perovskite with *ASD* = 0 and *μ* = 0 for the fully disordered double perovskite with *ASD* = 0.5. The linear decrease of the saturation magnetization of Sr_2_FeMoO_6_ with increasing *ASD* has also been predicted on the basis of Monte Carlo simulations (Ogale et al., 1999) and *ab initio* calculations (Reyes et al., 2016). The degradation of magnetic properties of Sr_2_FeMoO_6_ with increasing cation disorder has also been confirmed experimentally in numerous papers, see, e.g., (Suchaneck et al., 2020).

The magnetization hysteresis loops of the as-prepared nanostructured Sr_2_FeMoO_6_ measured at 300 and 10 K are depicted in Figure 7B. As expected for magnetization measurements above *T*_B_, the room-temperature hysteresis loop of the nanomaterial is dominated by the superparamagnetic relaxation effect, whereas a slight magnetic hysteresis is observed at 10 K. Thus, the room-temperature *M*(*H*_ext_) curve was analyzed in terms of superparamagnetic behavior using the Brillouin function. The effective magnetic moment derived from the analysis attains the value *μ* = 0.055 *μ*_B_/f.u. Using the above given relation *μ* = (4 – 8 *ASD*) *μ*_B_ and the experimentally determined magnetic moment *μ* = 0.055 *μ*_B_, the value of *ASD* for the as-prepared Sr_2_FeMoO_6_ is estimated to be about 0.493. This value derived from SQUID measurements is in excellent agreement with that obtained from the present Mössbauer and XRD data (see above). This extraordinarily high degree of anti-site disorder in the as-prepared nanostructured Sr_2_FeMoO_6_ corresponds to the completely random distribution of cations with a maximum configurational entropy. It should be emphasized that the nearly random distribution of cations has also been reported for spinel oxides prepared by mechanochemical routes (Šepelák et al., 2013).

## Conclusion

The high-energy milling of the 2 SrO + α-Fe + MoO_3_ mixture at ambient temperature in air leads to the mechanochemical synthesis of the tetragonal double perovskite Sr_2_FeMoO_6_ with the lattice parameters *a* = 5.5856(5) Å and *c* = 7.8970(140) Å. The overall reaction path leading to Sr_2_FeMoO_6_ involves the formation of the intermediate SrMoO_4_ ternary phase, and can be expressed by the following sequence of two mechanochemical reactions: 2 SrO + α-Fe^0^ + Mo^6+^O_3_ + ½ O_2_ →
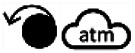
→ SrO + α-Fe^0^ + SrMo^6+^O_4_ + ½ O_2_ →
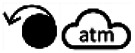
→ Sr_2_Fe^3+^Mo^5+^O_6_. The first one represents the mechanochemical synthesis of the intermediate SrMo^6+^O_4_ phase, which is not accompanied by the valence change of the constituent cations, whereas the subsequent mechanochemical redox reaction includes the reduction of Mo^6+^ cations and the oxidation of Fe^0^ atoms. This unique mechanochemical synthesis accompanied by such a large extent of oxidation (Fe^0^ → Fe^3+^) has not yet been reported. The degree of conversion of the present mechanochemical synthesis is estimated to be about 90%. The subsequent thermal treatment of the mechanically activated material at a fairly moderate temperature of 923 K for 30 min in air results in the full conversion of the reactants into the double perovskite Sr_2_FeMoO_6_ phase. Despite the latter processing, the completely synthesized Sr_2_FeMoO_6_ possesses the nanostructured nature characterized by a relatively broad particle size distribution (7–28 nm) with the average particle size of about 21(4) nm and the roughly spherical particle shape. The degree of anti-site disorder in the as-prepared Sr_2_FeMoO_6_ is found to be *ASD* = 0.5, demonstrating its far-from-equilibrium, highly disordered structural state possessing the completely random distribution of Fe^3+^ and Mo^5+^ cations over the sites of octahedral coordination provided by the double perovskite structure. It should be emphasized that this record-high value of *ASD* = 0.5, derived independently from the present experimental XRD, Mössbauer, and magnetization data, is, to the best of our knowledge, the highest value so far reported for the Sr_2_FeMoO_6_ phase. At the short-range scale, the iron nuclei located on the anti-sites ([Fe_Mo_]) experience a relatively broad distribution of magnetic hyperfine fields of about 10 T (from about 45 to 55 T) reflecting both, the presence of a wide variation in the local chemical environment around Fe^3+^ cations from site to site and the presence of a strongly distorted geometry of the constituent FeO_6_ octahedra. The nanostructured and fully anti-site disordered Sr_2_FeMoO_6_ exhibits the superparamagnetic behavior with the blocking temperature of about *T*_B_ = 240 K and the effective magnetic moment *μ* = 0.055 *μ*_B_/f.u.

## Data Availability

The original contributions presented in the study are included in the article/supplementary material, further inquiries can be directed to the corresponding author.

## References

[B1] Alvarado-Flores J. J. Mondragón-Sánchez R. Ávalos-Rodríguez M. L. Alcaraz-Vera J. V. Rutiaga-Quiñones J. G. Guevara-Martínez S. J. (2021). Synthesis, Characterization and Kinetic Study of the Sr_2_FeMoO_6-δ_ Double Perovskite: New Findings on the Calcination of One of its Precursors. Int. J. Hydrogen Energ. 46 (51), 26185–26196. 10.1016/j.ijhydene.2021.01.191

[B2] Amrute A. P. De Bellis J. Felderhoff M. Schüth F. (2021). Mechanochemical Synthesis of Catalytic Materials. Chem. Eur. J. 27 (23), 6819–6847. 10.1002/chem.202004583 33427335 PMC8248068

[B3] Andersen J. Mack J. (2018). Mechanochemistry and Organic Synthesis: From Mystical to Practical. Green. Chem. 20 (7), 1435–1443. 10.1039/c7gc03797j

[B4] Avvakumov G. V. Senna M. Kosova N. V. (2001). Soft Mechanochemical Synthesis: A Basis for New Chemical Technologies. Boston: Kluwer Academic Publishers.

[B5] Baláž P. Achimovičová M. Baláž M. Billik P. Cherkezova-Zheleva Z. Criado J. M. (2013). Hallmarks of Mechanochemistry: From Nanoparticles to Technology. Chem. Soc. Rev. 42 (18), 7571–7637. 10.1039/c3cs35468g 23558752

[B6] Bensch W. Breu J. (2017). Editorial. Z. Krist.-Cryst. Mater. 232 (1−3), 1. 10.1515/zkri-2017-5001

[B7] Boldyrev V. V. (2018). Mechanochemical Processes with the Reaction-Induced Mechanical Activation. Chemo-Mechanochemical Effect. Russ. Chem. Bull. 67 (6), 933–948. 10.1007/s11172-018-2162-z

[B8] Bolm C. Hernández J. G. (2018). From Synthesis of Amino Acids and Peptides to Enzymatic Catalysis: A Bottom-Up Approach in Mechanochemistry. ChemSusChem 11 (9), 1410–1420. 10.1002/cssc.201800113 29436773

[B9] Chmaissem O. Kruk R. Dabrowski B. Brown D. E. Xiong X. Kolesnik S. (2000). Structural Phase Transition and the Electronic and Magnetic Properties of Sr_2_FeMoO_6_ . Phys. Rev. B 62 (21), 14197–14206. 10.1103/PhysRevB.62.14197

[B10] Da Silva K. L. Menzel D. Feldhoff A. Kübel C. Bruns M. Paesano A. (2011). Mechanosynthesized BiFeO_3_ Nanoparticles with Highly Reactive Surface and Enhanced Magnetization. J. Phys. Chem. C 115 (15), 7209–7217. 10.1021/jp110128t

[B11] Da Silva K. L. Trautwein R. S. Da Silva R. B. Fabián M. Čižmár E. Holub M. (2021). Suppression of the Cycloidal Spin Arrangement in BiFeO_3_ Caused by the Mechanically Induced Structural Distortion and its Effect on Magnetism. Front. Mater. 8, 717185. 10.3389/fmats.2021.717185

[B12] De Oliveira P. F. M. Torresi R. M. Emmerling F. Camargo P. H. C. (2020). Challenges and Opportunities in the Bottom-Up Mechanochemical Synthesis of Noble Metal Nanoparticles. J. Mater. Chem. A. 8 (32), 16114–16141. 10.1039/d0ta05183g

[B13] El-Eskandarany M. S. Al-Hazza A. Al-Hajji L. A. Ali N. Al-Duweesh A. A. Banyan M. (2021). Mechanical Milling: A Superior Nanotechnological Tool for Fabrication of Nanocrystalline and Nanocomposite Materials. Nanomaterials 11 (10), 2484. 10.3390/nano11102484 34684925 PMC8539264

[B14] Farzin Y. A. Babaei A. Ataie A. (2020). Low-temperature Synthesis of Sr_2_FeMoO_6_ Double Perovskite; Structure, Morphology, and Magnetic Properties. Ceramics Int. 46 (10), 16867–16878. 10.1016/j.ceramint.2020.03.264

[B15] Friščić T. Mottillo C. Titi H. M. (2020). Mechanochemistry for Synthesis. Angew. Chem. Intl Edit 59 (3), 1018–1029. 10.1002/anie.201906755 31294885

[B16] Głowniak S. Szczęśniak B. Choma J. Jaroniec M. (2021). Mechanochemistry: Toward Green Synthesis of Metal-Organic Frameworks. Mater. Today 46, 109–124. 10.1016/j.mattod.2021.01.008

[B31] Godočíková E. Baláž P. Boldižárová E. Škorvánek I. Kováč J. Choi W. (2004). Mechanochemical Reduction of Lead Sulphide by Elemental Iron. J. Mater. Sci. 39 (16-17), 5353–5355. 10.1023/B:JMSC.0000039243.89535.ef

[B17] Gombotz M. Wilkening H. M. R. (2021). Fast Li Ion Dynamics in the Mechanosynthesized Nanostructured Form of the Solid Electrolyte Li_3_YBr_6_ . ACS Sust. Chem. Eng. 9 (2), 743–755. 10.1021/acssuschemeng.0c06694

[B18] Gomollón-Bel F. (2019). Ten Chemical Innovations that Will Change Our World: IUPAC Identifies Emerging Technologies in Chemistry with Potential to Make Our Planet More Sustainable. Chem. Int. 41 (2), 12–17. 10.1515/ci-2019-0203

[B19] Greneche J. M. Venkatesan M. Suryanarayanan R. Coey J. M. D. (2001). Mössbauer Spectrometry of A_2_FeMoO_6_ (A = Ca, Sr, Ba): Search for Antiphase Domains. Phys. Rev. B 63 (17), 174403. 10.1103/PhysRevB.63.174403

[B20] Hansen M. F. Mørup S. (1999). Estimation of Blocking Temperatures from ZFC/FC Curves. J. Magn. Magn. Mater. 203 (1-3), 214–216. 10.1016/S0304-8853(99)00238-3

[B21] Harris V. G. Šepelák V. (2018). Mechanochemically Processed Zinc Ferrite Nanoparticles: Evolution of Structure and Impact of Induced Cation Inversion. J. Magnetism Magn. Mater. 465, 603–610. 10.1016/j.jmmm.2018.05.100

[B22] Huan Y. Li Y. Yin B. Ding D. Wei T. (2017). High Conductive and Long-Term Phase Stable Anode Materials for SOFCs: A_2_FeMoO_6_ (A = Ca, Sr, Ba). J. Power Sourc. 359, 384–390. 10.1016/j.jpowsour.2017.05.079

[B23] Huang Y. H. Lindén J. Yamauchi H. Karppinen M. (2004). Simple and Efficient Route to Prepare Homogeneous Samples of Sr_2_FeMoO_6_ with a High Degree of Fe/Mo Order. Chem. Mater. 16 (22), 4337–4342. 10.1021/cm0493288

[B24] Iranmanesh M. Lingg M. Stir M. Hulliger J. (2016). Sol Gel and Ceramic Synthesis of Sr_2_FeMo_1−x_W_x_O_6_ (0 ≤ x ≤ 1) Double Perovskites Series. RSC Adv. 6 (48), 42069–42075. 10.1039/c6ra03923e

[B25] Jalili H. Heinig N. F. Leung K. T. (2009). X-ray Photoemission Study of Sr_2_FeMoO_6_ and SrMoO_4_ Films Epitaxially Grown on MgO(001): Near-Surface Chemical-State Composition Analysis. Phys. Rev. B 79 (17), 174427. 10.1103/PhysRevB.79.174427

[B26] Kalanda N. Kim D.-H. Demyanov S. Yu S.-C. Yarmolich M. Petrov A. (2018). Sr_2_FeMoO_6_ Nanosized Compound with Dielectric Sheaths for Magnetically Sensitive Spintronic Devices. Curr. Appl. Phys. 18 (1), 27–33. 10.1016/j.cap.2017.10.018

[B27] Kalanda N. Turchenko V. Karpinsky D. Demyanov S. Yarmolich M. Balasoiu M. (2019). The Role of the Fe/Mo Cations Ordering Degree and Oxygen Non‐Stoichiometry on the Formation of the Crystalline and Magnetic Structure of Sr_2_FeMoO_6−δ_ . Phys. Status Solidi B 256 (5), 1800278. 10.1002/pssb.201800278

[B28] Kalanda N. Yarmolich M. Petrov A. Raevski I. Kubrin S. Raevskaya S. (2020). The Influence of Cation Ordering and Oxygen Nonstoichiometry on Magnetic Properties of Sr_2_FeMoO_6−x_ Around Curie Temperature. J. Magnetism Magn. Mater. 500, 166386. 10.1016/j.jmmm.2019.166386

[B29] Kobayashi K.-I. Kimura T. Sawada H. Terakura K. Tokura Y. (1998). Room-Temperature Magnetoresistance in an Oxide Material with an Ordered Double-Perovskite Structure. Nature 395 (6703), 677–680. 10.1038/27167

[B30] Kobayashi M. Tanaka K. Fujimori A. Ray S. Sarma D. D. (2007). Critical Test for Altshuler-Aronov Theory: Evolution of the Density of States Singularity in Double Perovskite Sr_2_FeMoO_6_ with Controlled Disorder. Phys. Rev. Lett. 98 (24), 246401. 10.1103/PhysRevLett.98.246401 17677976

[B32] Lapshin O. V. Boldyreva E. V. Boldyrev V. V. (2021). Role of Mixing and Milling in Mechanochemical Synthesis (Review). Russ. J. Inorg. Chem. 66 (3), 433–453. 10.1134/S0036023621030116

[B33] Li X. Y. Yao Z. F. Zhang L. Y. Zheng G. H. Dai Z. X. Chen K. Y. (2019). Generation of Oxygen Vacancies on Sr_2_FeMoO_6_ to Improve its Photocatalytic Performance through a Novel Preparation Method Involving pH Adjustment and Use of Surfactant. Appl. Surf. Sci. 480, 262–275. 10.1016/j.apsusc.2019.02.115

[B34] Lindén J. Yamamoto T. Karppinen M. Yamauchi H. Pietari T. (2000). Evidence for Valence Fluctuation of Fe in Sr_2_FeMoO_6−w_ Double Perovskite. Appl. Phys. Lett. 76 (20), 2925–2927. 10.1063/1.126518

[B35] Long G. J. (1987). Mössbauer Spectroscopy Applied to Inorganic Chemistry. New York: Plenum Press.

[B36] Lunghammer S. Düvel A. Posch P. Kunert B. Resel R. Wilkening H. M. R. (2019). Self-diffusion and Ionic Exchange in Mechanosynthesized, Nanocrystalline Solid Solutions of PbF_2_ and CaF_2_ – ^19^F 2D NMR Visualizes the Flourine Hopping Preferences. Solid State Ionics 343, 115067. 10.1016/j.ssi.2019.115067

[B37] Maiti P. Guha P. Hussain H. Singh R. Nicklin C. Satyam P. V. (2019). Microscopy and Spectroscopy Study of Nanostructural Phase Transformation from β-MoO_3_ to Mo under UHV - MBE Conditions. Surf. Sci. 682, 64–74. 10.1016/j.susc.2018.12.008

[B38] Menéndez N. García-Hernández M. Sánchez D. Tornero J. D. Martínez J. L. Alonso J. A. (2004). Charge Transfer and Disorder in Double Perovskites. Chem. Mater. 16 (18), 3565–3572. 10.1021/cm049305t

[B39] Menil F. (1985). Systematic Trends of the ^57^Fe Mössbauer Isomer Shifts in (FeO_n_) and (FeF_n_) Polyhedra. Evidence of a New Correlation between the Isomer Shift and the Inductive Effect of the Competing Bond T-X (→ Fe) (Where X Is O or F and T Any Element with a Formal Positive Charge). J. Phys. Chem. Sol. 46 (7), 763–789. 10.1016/0022-3697(85)90001-0

[B40] Menzel M. Šepelák V. Becker K. D. (2001). Mechanochemical Reduction of Nickel Ferrite. Solid State Ionics 141-142, 663–669. 10.1016/S0167-2738(01)00802-5

[B41] Mestl G. Ruiz P. Delmon B. Knozinger H. (1994). Oxygen-Exchange Properties of MoO_3_: An *In Situ* Raman Spectroscopy Study. J. Phys. Chem. 98 (44), 11269–11275. 10.1021/j100095a007

[B42] Michalchuk A. A. L. Boldyreva E. V. Belenguer A. M. Emmerling F. Boldyrev V. V. (2021). Tribochemistry, Mechanical Alloying, Mechanochemistry: What Is in a Name? Front. Chem. 9, 685789. 10.3389/fchem.2021.685789 34164379 PMC8216082

[B43] Michalchuk A. A. L. Tumanov I. A. Boldyreva E. V. (2019). Ball Size or Ball Mass - what Matters in Organic Mechanochemical Synthesis? CrystEngComm 21 (13), 2174–2179. 10.1039/c8ce02109k

[B44] Mon J. P. (1972). Spectres de diffusion Raman de l'oxyde de strontium. J. Phys. Chem. Sol. 33 (6), 1257–1260. 10.1016/S0022-3697(72)80164-1

[B45] Nakamura S. Oikawa K. (2003). Precise Structure Analysis Consistent with Mössbauer Quadrupole Effect: A Case of the Ordered Double Perovskites Sr_2_FeMO_6_ (M = Mo and Re). J. Phys. Soc. Jpn. 72 (12), 3123–3127. 10.1143/JPSJ.72.3123

[B46] Navarro J. Frontera C. Rubi D. Mestres N. Fontcuberta J. (2003). Aging of Sr_2_FeMoO_6_ and Related Oxides. Mater. Res. Bull. 38 (9-10), 1477–1486. 10.1016/S0025-5408(03)00171-5

[B47] Néel L. (1949). Théorie du traînage magnétique des ferromagnétiques en grains fins avec application aux terres cuites. Ann. de géophysique 5, 99–136.

[B48] Ogale A. S. Ogale S. B. Ramesh R. Venkatesan T. (1999). Octahedral Cation Site Disorder Effects on Magnetization in Double-Perovskite Sr_2_FeMoO_6_: Monte Carlo Simulation Study. Appl. Phys. Lett. 75 (4), 537–539. 10.1063/1.124440

[B49] O’Neill R. T. Boulatov R. (2021). The Many Flavours of Mechanochemistry and its Plausible Conceptual Underpinnings. Nat. Rev. Chem. 5 (3), 148–167. 10.1038/s41570-020-00249-y 37117533

[B50] Pickhardt W. Grätz S. Borchardt L. (2020). Direct Mechanocatalysis: Using Milling Balls as Catalysts. Chem. Eur. J. 26 (57), 12903–12911. 10.1002/chem.202001177 32314837 PMC7589287

[B51] Porcheddu A. Colacino E. De Luca L. Delogu F. (2020). Metal-Mediated and Metal-Catalyzed Reactions under Mechanochemical Conditions. ACS Catal. 10 (15), 8344–8394. 10.1021/acscatal.0c00142

[B52] Porodko O. Fabián M. Kolev H. Lisnichuk M. Zukalová M. Vinarčíková M. (2021). A Novel High Entropy Spinel-type Aluminate MAl_2_O_4_ (M = Zn, Mg, Cu, Co) and its Lithiated Oxyfluoride and Oxychloride Derivatives Prepared by One-step Mechanosynthesis. Z. Phys. Chem., 000010151520213106. 10.1515/zpch-2021-3106

[B53] Putz H. Brandenburg K. (2019). Diamond – Crystal and Molecular Structure Visualization. Version 4.6. Bonn: Crystal Impact.

[B54] Reyes A. M. Arredondo Y. Navarro O. (2016). Effect of Cationic Disorder on the Magnetic Moment of Sr_2_FeMoO_6_: Ab Initio Calculations. J. Phys. Chem. C 120 (7), 4048–4052. 10.1021/acs.jpcc.6b00100

[B56] Saloaro M. Hoffmann M. Adeagbo W. A. Granroth S. Deniz H. Palonen H. (2016). Toward Versatile Sr_2_FeMoO_6_-Based Spintronics by Exploiting Nanoscale Defects. ACS Appl. Mater. Inter. 8 (31), 20440–20447. 10.1021/acsami.6b04132 27447197

[B57] Sarma D. D. Mahadevan P. Saha-Dasgupta T. Ray S. Kumar A. (2000b). Electronic Structure of Sr_2_FeMoO_6_ . Phys. Rev. Lett. 85 (12), 2549–2552. 10.1103/PhysRevLett.85.2549 10978104

[B58] Sarma D. D. Sampathkumaran E. V. Ray S. Nagarajan R. Majumdar S. Kumar A. (2000a). Magnetoresistance in Ordered and Disordered Double Perovskite Oxide, Sr_2_FeMoO_6_ . Solid State. Commun. 114 (9), 465–468. 10.1016/S0038-1098(00)00079-X

[B59] Schlem R. Burmeister C. F. Michalowski P. Ohno S. Dewald G. F. Kwade A. (2021). Energy Storage Materials for Solid‐State Batteries: Design by Mechanochemistry. Adv. Energ. Mater. 11 (30), 2101022. 10.1002/aenm.202101022

[B60] Šepelák V. Becker K. D. Bergmann I. Suzuki S. Indris S. Feldhoff A. (2009). A One-step Mechanochemical Route to Core−Shell Ca_2_SnO_4_ Nanoparticles Followed by ^119^Sn MAS NMR and ^119^Sn Mössbauer Spectroscopy. Chem. Mater. 21 (12), 2518–2524. 10.1021/cm900590d

[B61] Šepelák V. Becker K. D. (2000). Mössbauer Studies in the Mechanochemistry of Spinel Ferrites. J. Mater. Synth. Process. 8 (3-4), 155–166. 10.1023/A:1011355908538

[B62] Šepelák V. Bégin-Colin S. Le Caër G. (2012a). Transformations in Oxides Induced by High-Energy Ball-Milling. Dalton Trans. 41 (39), 11927–11948. 10.1039/c2dt30349c 22875201

[B63] Šepelák V. Bergmann I. Feldhoff A. Heitjans P. Krumeich F. Menzel D. (2007). Nanocrystalline Nickel Ferrite, NiFe_2_O_4_: Mechanosynthesis, Nonequilibrium Cation Distribution, Canted Spin Arrangement, and Magnetic Behavior. J. Phys. Chem. C 111 (13), 5026–5033. 10.1021/jp067620s

[B64] Šepelák V. Da Silva K. L. Trautwein R. S. Becker K. D. Hahn H. (2021). Unusual Cation Coordination in Nanostructured Mullites. Z. Phys. Chem., 000010151520213101. 10.1515/zpch-2021-3101

[B65] Šepelák V. Düvel A. Wilkening M. Becker K.-D. Heitjans P. (2013). Mechanochemical Reactions and Syntheses of Oxides. Chem. Soc. Rev. 42 (18), 7507–7520. 10.1039/c2cs35462d 23364473

[B66] Šepelák V. Feldhoff A. Heitjans P. Krumeich F. Menzel D. Litterst F. J. (2006). Nonequilibrium Cation Distribution, Canted Spin Arrangement, and Enhanced Magnetization in Nanosized MgFe_2_O_4_ Prepared by a One-step Mechanochemical Route. Chem. Mater. 18 (13), 3057–3067. 10.1021/cm0514894

[B67] Šepelák V. Menzel M. Becker K. D. Krumeich F. (2002). Mechanochemical Reduction of Magnesium Ferrite. J. Phys. Chem. B 106 (26), 6672–6678. 10.1021/jp020270z

[B68] Šepelák V. Myndyk M. Fabián M. Silva K. L. D. Feldhoff A. Menzel D. (2012b). Mechanosynthesis of Nanocrystalline Fayalite, Fe_2_SiO_4_ . Chem. Commun. 48 (90), 11121–11123. 10.1039/c2cc36370d 23042410

[B69] Shimada T. Nakamura J. Motohashi T. Yamauchi H. Karppinen M. (2003). Kinetics and Thermodynamics of the Degree of Order of the B Cations in Double-Perovskite Sr_2_FeMoO_6_ . Chem. Mater. 15 (23), 4494–4497. 10.1021/cm030409y

[B70] Skurikhina O. Senna M. Fabián M. Witte R. Tarasenko R. Tkáč V. (2020). A Sustainable Reaction Process for Phase Pure LiFeSi_2_O_6_ with Goethite as an Iron Source. Ceramics Int. 46 (10), 14894–14901. 10.1016/j.ceramint.2020.03.016

[B71] Skutina L. Filonova E. Medvedev D. Maignan A. (2021). Undoped Sr_2_MMoO_6_ Double Perovskite Molybdates (M = Ni, Mg, Fe) as Promising Anode Materials for Solid Oxide Fuel Cells. Materials 14 (7), 1715. 10.3390/ma14071715 33807360 PMC8036809

[B72] Solares-Briones M. Coyote-Dotor G. Páez-Franco J. C. Zermeño-Ortega M. R. de la O Contreras C. M. Canseco-González D. (2021). Mechanochemistry: A Green Approach in the Preparation of Pharmaceutical Cocrystals. Pharmaceutics 13 (6), 790. 10.3390/pharmaceutics13060790 34070646 PMC8228148

[B73] Son L. H. Phuc N. X. Phuc P. V. Hong N. M. Hong L. V. (2001). Observation of Phase Decomposition of Sr_2_FeMoO_6_ by Raman Spectroscopy. J. Raman Spectrosc. 32 (10), 817–820. 10.1002/jrs.764

[B74] Suchaneck G. Kalanda N. Artsiukh E. Gerlach G. (2020). Challenges in Sr_2_FeMoO_6−δ_ Thin Film Deposition. Phys. Status Solidi B 257 (3), 1900312. 10.1002/pssb.201900312

[B75] Sujatha R. A. Flower N. A. L. Vinitha G. Sharath R. A. Rahulan K. M. (2019). Structural and Non-linear Optical Response of Er^3+^ Doped SrMoO_4_ Nanostructures. Appl. Surf. Sci. 490, 260–265. 10.1016/j.apsusc.2019.06.086

[B76] Suominen T. Raittila J. Salminen T. Schlesier K. Lindén J. Paturi P. (2007). Magnetic Properties of Fine SFMO Particles: Superparamagnetism. J. Magnetism Magn. Mater. 309 (2), 278–284. 10.1016/j.jmmm.2006.07.016

[B77] Szczęśniak B. Choma J. Jaroniec M. (2021). Recent Advances in Mechanochemical Synthesis of Mesoporous Metal Oxides. Mater. Adv. 2 (8), 2510–2523. 10.1039/d1ma00073j

[B78] Tóthová E. Tarasenko R. Tkáč V. Orendáč M. Hegedüs M. Danková Z. (2019). Microcrystalline Gd_2_MoO_6_ Prepared by Combined Mechanochemical/Thermal Process and its Magnetic Properties. J. Mater. Sci. 54 (8), 6111–6121. 10.1007/s10853-019-03331-z

[B79] Tóthová E. Witte R. Hegedüs M. Senna M. Hahn H. Heitjans P. (2018). Mechanochemical Syntheses of LiFeGe_2_O_6_-Based Nanocomposite and Novel Nanoglassy LiFeTi_2_O_6_ . J. Mater. Sci. 53 (19), 13530–13537. 10.1007/s10853-018-2405-2

[B80] Tsuzuki T. (2021). Mechanochemical Synthesis of Metal Oxide Nanoparticles. Commun. Chem. 4 (1), 143. 10.1038/s42004-021-00582-3 PMC9814100 36697599

[B81] Valdés J. Reséndiz D. Cuán Á. Nava R. Aguilar B. Cortés-Romero C. M. (2021). Sol-Gel Synthesis of the Double Perovskite Sr_2_FeMoO_6_ by Microwave Technique. Materials 14 (14), 3876. 10.3390/ma14143876 34300797 PMC8305548

[B82] Valenzuela J. L. Soto T. E. Lemus J. Navarro O. Morales R. (2014). Reaction Kinetics of the Double Perovskite Sr_2_FeMoO_6_ by Gas-Solid Reactions. Physica B: Condensed Matter 455, 10–13. 10.1016/j.physb.2014.07.034

[B83] Wilkening M. Düvel A. Preishuber-Pflügl F. Da Silva K. Breuer S. Šepelák V. (2017). Structure and Ion Dynamics of Mechanosynthesized Oxides and Fluorides. Z. Krist.-Cryst. Mater. 232 (1−3), 107–127. 10.1515/zkri-2016-1963

[B84] Xi X. Liu J. Fan Y. Wang L. Li J. Li M. (2021). Reducing d-p Band Coupling to Enhance CO_2_ Electrocatalytic Activity by Mg-Doping in Sr_2_FeMoO_6−δ_ Double Perovskite for High Performance Solid Oxide Electrolysis Cells. Nano Energy 82, 105707. 10.1016/j.nanoen.2020.105707

[B85] Yarmolich M. Kalanda N. Demyanov S. Fedotova J. Bayev V. Sobolev N. A. (2016). Charge Ordering and Magnetic Properties in Nanosized Sr_2_FeMoO_6−δ_ Powders. Phys. Status Solidi B 253 (11), 2160–2166. 10.1002/pssb.201600527

[B86] Yu S. C. Song Y. Y. Kiss L. F. Vincze I. (1999). Study on the Magnetic Behavior of Nanogranular Cu_80_Fe_10_Co_10_ Solid Solution. J. Magn. Magn. Mater. 203 (1-3), 316–318. 10.1016/S0304-8853(99)00214-0

[B87] Zhang Q. Saito F. (2012). A Review on Mechanochemical Syntheses of Functional Materials. Adv. Powder Tech. 23 (5), 523–531. 10.1016/j.apt.2012.05.002

[B88] Zhou X. Miao Y.-R. Shaw W. L. Suslick K. S. Dlott D. D. (2019). Shock Wave Energy Absorption in Metal-Organic Framework. J. Am. Chem. Soc. 141 (6), 2220–2223. 10.1021/jacs.8b12905 30700090

